# Editorial: Lymphatic system: organ specific functions in health and disease

**DOI:** 10.3389/fcell.2023.1224584

**Published:** 2023-10-23

**Authors:** Kaska Koltowska, Zoltán Jakus, Young-Kwon Hong, Tsutomu Kume

**Affiliations:** ^1^ Department of Immunology, Genetics and Pathology, Uppsala University, Uppsala, Sweden; ^2^ Beijer Gene and Neuro Laboratory and Science for Life Laboratories, Uppsala University, Uppsala, Sweden; ^3^ Department of Physiology, Semmelweis University, Budapest, Hungary; ^4^ Keck School of Medicine, University of Southern California, Los Angeles, CA, United States; ^5^ Northwestern University Feinberg School of Medicine, Chicago, IL, United States

**Keywords:** adipose tissue lymphatics, volumetric imaging, lymph filtering, organotypic lymphatic vascular, lymphatic vessel biology

## Introduction

The lymphatic system is composed of a lymphatic vascular network and lymph nodes, lymphoid organs, lymphoid tissue and a colorless fluid called lymph. The lymphatic vasculature is a vital component of our bodies and upholds crucial physiological functions. This intricate network of vessels is responsible for lymph drainage to maintain fluid balance. It is essential for immune cell trafficking to support immune functions and is involved in lipid absorption and transport from the gut. Lymph nodes play a pivotal role in the initiation of immune responses by detecting microbial components and foreign substances present in the lymph. The largest lymphoid organ is the spleen which is involved in immune responses and in filtering the blood. Dysfunction of the lymphatic system can lead to a variety of diseases and conditions, such as lymphedema, cancer metastasis, and inflammation. Recent research has revealed organ-specific functions of the lymphatic system and their implications for health and disease (Petrova and Koh, 2018; Oliver et al., 2020).

In a focused exploration of the organ-specific functions of the lymphatic system, the Research Topic “*Lymphatic system: Organ specific functions in health and disease*,” brought together a Research Topic of five original research articles and three review articles. These offer valuable insights into the lymphatic field and highlight the recent advances in our understanding of lymphatic system formation and function. The contributing articles in this Research Topic cover a broad range of topics related to the lymphatic system. Focusing on visualization and formation of lymphatic vasculature in different organs including the liver, adipose tissue, spleen, and central nervous system (CNS). Highlighting new mechanisms of filtering in lymph nodes and the significance of fluid pressure in cancer. Finally, the Research Topic includes studies discussing the relationship between gene mutations and fetal oedema and modulation of lymphatic valve number in the hope of new avenues for improving lymphatic vessel function.

## Organ-specific lymphatic vasculature

Volumetric Imaging of Hepatic Lymph Vessels (Bobe et al.). The liver, a central organ in metabolism and detoxification, has been underexplored for its role in lymph generation and composition. Bobe and his team employed cutting-edge volumetric imaging to unveil the microarchitecture of hepatic lymphatic vessels. They discovered a unique lymphatic network exclusively located within the portal tracts of the liver, which plays a crucial role in lymph flow. This study provides essential insights into liver physiology and lymphatics, highlighting their interdependence.

Adipose Tissue Lymphatics and Thermogenesis (Phan et al.). This study uncovered a surprising link between adipose tissue lymphatic vessels and thermogenesis regulation. Researchers have found that adipose tissue lymphatics are a significant source of the neuropeptide neurotensin, which inhibits thermogenesis. By inducing lymphangiogenesis, they impair thermogenic capacity in adipose tissue, shedding light on the neural and hormonal regulation of body temperature.

Nkx2-3 and Splenic Microvascular Patterning Gabris et al. delved into the intricate microvascular architecture of the spleen and its role in hematopoiesis. They identified how the absence of the Nkx2-3 gene led to the emergence of ectopic lymphatic vessels in the spleen, which disrupted splenic erythropoiesis and megakaryocyte colony formation. This study highlights the critical role of Nkx2-3 in maintaining vascular specification and its impact on hematopoietic function in the spleen.

Lymphatic Vasculature in the CNS. Gonzalez-Hernandez and Mukouyama discussed the long-standing notion of the central nervous system as an immune-privileged organ and explored the recent discovery of lymphatic structures in the CNS.

These articles provide insights into the unique functions of lymphatic vessels in different organs and tissues and how they contribute to maintaining body homeostasis.

## Lymph filtering and fluid pressure

Micro- and Macro-Anatomical Frameworks of Lymph Nodes Ozawa et al. explored the intricate anatomy and functionality of lymph nodes (LNs) within the lymphatic system. They discovered a critical role for resident macrophages and specialized lymphatic sinus structures in filtering lymph-borne substances. Additionally, their research highlighted the stepwise filtration process orchestrated by lymph nodes in preventing the entry of foreign substances into the bloodstream. The study demonstrated the dynamic and organized nature of lymphatic recirculation and its adaptability during inflammatory responses.

The Ambivalent Nature of the Lymphatics-Cancer Relationship Choi et al. provided a comprehensive overview of the complex interplay between lymphatic vessels, cancer, and tumor fluid pressure, suggesting potential applications of lymphatics in antitumor therapy.

These articles highlight the importance of constituting a filtering system for lymphatic recirculation and demonstrate lymphatic vessels’ crucial function in immune surveillance and fluid pressure drainage in the tumor microenvironment.

## Lymphedema causes and hopes

Fetal Nuchal Edema and Gene Mutations. Sugiyama and Hirashima reviewed the relationship between gene mutants causing fetal nuchal edema and various developmental anomalies in mice, shedding light on potential diagnostic and prognostic applications.

Pharmacological Inhibition of FOXO1 in Lymphatic Valve Growth (Ogunsina et al.). Mutations in genes governing lymphatic valve development are associated with congenital lymphedema. Investigated the potential of a FOXO1 inhibitor to stimulate lymphatic valve formation. Their research demonstrated the ability of this inhibitor to upregulate valve-forming genes in lymphatic endothelial cells. Furthermore, in mouse models, the inhibition of FOXO1 led to a significant increase in lymphatic valve numbers, offering a potential avenue to improve lymphatic vessel function.

These articles shed light on the molecular and cellular mechanisms underlying lymphatic vascular development and explore potential therapeutic strategies for lymphatic-related disorders.

In conclusion, the articles on this Research Topic provide a comprehensive overview of the organ-specific functions of the lymphatic system in health and disease. They showcase exciting advances in our understanding of the lymphatic system and highlight the complexity of organotypic lymphatic vascular morphogenesis and function. However, how the underlying molecular and cellular heterogeneity is orchestrated into these different functions is less understood. Specifically, the extent of the interplay between the local microenvironment and lymphatic vessels is needed to achieve functional heterogeneity.

Overall, this Research Topic depicted the importance of the lymphatic system in maintaining homeostasis and fighting diseases. It also emphasizes the need for further research into the lymphatic system to develop effective diagnostic and therapeutic approaches for lymphatic-related disorders. We hope that this Research Topic will stimulate further research and discussion on the lymphatic system and its role in human health.
